# Comparative Postmarket Safety Profile of Adjuvanted and High-Dose Influenza Vaccines in Individuals 65 Years or Older

**DOI:** 10.1001/jamanetworkopen.2020.4079

**Published:** 2020-05-05

**Authors:** Alexis J. Pillsbury, Parveen Fathima, Helen E. Quinn, Patrick Cashman, Christopher C. Blyth, Alan Leeb, Kristine K. Macartney

**Affiliations:** 1 National Centre for Immunisation Research and Surveillance, The Children’s Hospital at Westmead, Westmead, Australia; 2 Faculty of Health and Medicine, University of Sydney, Camperdown, Australia; 3 Wesfarmers Centre of Vaccines and Infectious Diseases, Telethon Kids Institute, Perth, Australia; 4 Hunter New England Population Health, Newcastle, Australia; 5 School of Medicine, University of Western Australia, Perth, Australia; 6 Perth Children’s Hospital, Perth, Australia; 7 Department of Microbiology, PathWest Laboratory Medicine, QEII Medical Centre, Perth, Australia; 8 SmartVax, Illawarra Medical Centre, Ballajura, Australia

## Abstract

**Importance:**

Every year, influenza vaccines are administered to millions of people worldwide to reduce morbidity and mortality from influenza. As new vaccine formulations are increasingly used, monitoring and comparing safety, in addition to vaccine effectiveness, in target populations are essential.

**Objective:**

To assess the postmarketing safety profile of 2018 Southern Hemisphere influenza vaccines, particularly 2 new enhanced trivalent inactivated influenza vaccines: an adjuvanted trivalent inactivated influenza vaccine (aIIV3) and high-dose trivalent inactivated influenza vaccine (HD-IIV3), among Australian individuals 65 years or older.

**Design, Setting, and Participants:**

This cohort study used solicited short message service–based self-reported survey data on adverse events occurring within 3 to 5 days after receipt of an influenza vaccination. Participants included individuals 65 years or older who received routinely recommended influenza vaccines at 1 of 265 sentinel immunization sites, including primary care, hospital, and community-based clinics, participating in Australia’s AusVaxSafety active vaccine safety surveillance system from April 1 to August 31, 2018. Data were analyzed from September 1, 2018, to June 30, 2019.

**Exposure:**

Any licensed 2018 influenza vaccine administered in clinical practice.

**Main Outcomes and Measures:**

Rates (overall, by brand, and by concomitant vaccine receipt) of adverse events, including medical attendance as a proxy for serious adverse events.

**Results:**

Of 72 013 individuals 65 years or older who received an influenza vaccine in 2018, 50 134 individuals (69.6%) responded to the initial survey regarding adverse events experienced after vaccination (median [interquartile range] age, 71 [68-76] years; 27 056 [54.0%] women). Most individuals received an enhanced trivalent inactivated influenza vaccine, including 28 003 individuals (55.9%) who received aIIV3 and 19 306 individuals (38.5%) who received HD-IIV3; 2208 individuals (4.4%) received a quadrivalent inactivated influenza vaccine. Individuals who received HD-IIV3 reported significantly higher rates of any adverse event compared with individuals who received aIIV3 (1716 individuals [8.9%] vs 1796 individuals [6.4%]; P < .001) as well as specific adverse events, including fever (195 individuals [1.1%] vs 164 individuals [0.6%]; *P* < .001), injection site pain (383 individuals [2.1%] vs 350 individuals [1.3%]; *P* < .001), and injection site swelling or redness (256 individuals [1.4%] vs 248 individuals [0.9%]; *P* < .001). Adverse event rates reported by those receiving any quadrivalent inactivated influenza vaccine were similar to rates reported by those receiving aIIV3. Rates of medical care seeking for adverse events associated with aIIV3 and HD-IIV3 were low and comparable (80 individuals [0.3%] vs 56 individuals [0.3%]; *P* = .91).

**Conclusions and Relevance:**

The findings of this large-scale participant-based postmarketing assessment of the safety of 2 new enhanced influenza vaccines used in individuals 65 years or older provide reassuring near-real-time and cumulative data to inform and support confidence in ongoing vaccine use.

## Key Points

**Question:**

Were the 2018 adjuvanted trivalent inactivated influenza vaccine (TIIV) and high-dose TIIV associated with adverse events in Australian individuals 65 years or older?

**Findings:**

In this cohort study of active safety surveillance system data including 50 134 adults 65 years or older who responded to a short messaging service–based survey of self-reported adverse events following immunization, 94.4% of participants received an adjuvanted TIIV or high-dose TIIV. Individuals who received a high-dose TIIV reported higher rates of select adverse events, including fever and injection site reactions, but rates of seeking care were low and did not differ between enhanced vaccine groups.

**Meaning:**

The findings of this postmarketing active sentinel surveillance system study consisting of self-reported adverse events following immunization suggest that these 2 new enhanced influenza vaccines were well tolerated among an older Australian population.

## Teaser

This cohort study examines rates of adverse events following vaccinations among Australian adults 65 years or older who received influenza vaccines in 2018.

## Introduction

Globally, influenza kills hundreds of thousands of people each year,^1^ disproportionally affecting older individuals.^2^ Annual vaccination is the most effective public health measure available for influenza prevention, yet Australian and global coverage remain suboptimal.^3,4^ Following a severe influenza season in 2017 during which more than 90% of 1100 recorded influenza-related deaths occurred among individuals 65 years or older,^5^ the Australian National Immunisation Program (NIP) funded and preferentially recommended 2 new enhanced influenza vaccines for individuals 65 years or older: the non-adjuvanted high-dose trivalent inactivated influenza vaccine (TIIV), HD-IIV3, containing 60 µg of haemagglutinin per strain, and the adjuvanted TIIV, aIIV3, containing 15 µg of haemagglutinin per strain and MF59 oil in water emulsion adjuvant, rather than standard-dose quadrivalent inactivated influenza vaccine (QIIV).^6^ These represented the first new formulations, other than standard-dose inactivated influenza vaccine, used under the Australian NIP.^7^ Clinical trials and postlicensure studies have shown that both vaccines induce superior immunogenicity and offer improved effectiveness in older adults compared with standard TIIVs.^8,9,10,11,12,13,14^ While safety analyses from clinical trials^8,9,15,16^ have demonstrated increased injection site reaction rates for both enhanced vaccines compared with standard comparator TIIVs, reports of serious adverse events (SAEs) were uncommon.^9,14,15,16^

Because clinical trial safety data have inherent limitations,^17^ postmarketing vaccine safety surveillance serves as a critical adjunct, demonstrating how a vaccine is tolerated in real-world use among large populations. This is particularly important for influenza vaccines. In anticipation of each Northern and Southern Hemisphere influenza season, influenza vaccine strain composition may vary to target the predominant circulating viral strains.^7,17,18^ The variety of new influenza vaccine types available and manufacturing techniques used underpins the need to conduct additional postmarketing safety surveillance that can provide more detailed and timely brand-specific safety data than traditional spontaneous reporting systems can. To date, there are limited postmarketing safety data on high-dose and adjuvanted vaccines used in individuals 65 years or older. Surveillance that can monitor each season’s new influenza vaccines to rapidly identify potential safety issues, including by brand, is recommended.^17^ Safety incidents restricted to even 1 vaccine brand, such as the 2010 increase in severe febrile events in young Australian children,^19,20^ can result in program suspension and setbacks to vaccine confidence and overall influenza vaccine coverage.^21,22^ Numerous studies have demonstrated that concerns regarding influenza vaccine safety continue to contribute to hesitancy and poor vaccine uptake.^4,23,24^

Improvements to Australia’s vaccine safety surveillance following the 2010 safety incident and subsequent child influenza vaccination program suspension included creation of a national, active, sentinel surveillance system based on solicited participant-reported adverse events following immunization relayed via short message service (SMS). The AusVaxSafety vaccine safety surveillance system was established in 2014 and initially monitored influenza vaccine safety in children younger than 5 years, expanding to all ages and multiple vaccines from 2017.^25,26,27^ Throughout the 2018 influenza vaccination period (April to August 2018), data were available daily, with weekly reporting of both adverse event rates (by age, brand, and dose) and automated Bayesian signal detection^25^; no safety concerns were reported. Weekly analyses were reported to key immunization program stakeholders and the public via the AusVaxSafety.^28^ Using 2018 cumulative surveillance data, this study assessed the comparative safety profiles of the 2 new seasonal influenza vaccines, HD-IIV3 and aIIV3, used in Australians 65 years or older.

## Methods

AusVaxSafety and its surveillance tools operate nationally under human research ethical approval obtained from the Sydney Children’s Hospital Network and the Royal Australian College of General Practitioners National Research and Evaluation Ethics Committee. Participant consent is on an opt-out basis. This study is reported following the Strengthening the Reporting of Observational Studies in Epidemiology (STROBE) reporting guideline.

### Setting and Study Cohort

Australia’s population is approximately 25 million people.^29^ In 2018, AusVaxSafety captured data from more than 220 000 influenza vaccination encounters administered to individuals 6 months or older as routine clinical care at participating sentinel immunization sites, including general or family practices, hospitals, community-based immunization clinics, and Aboriginal Medical Services. In 2018, an estimated 11 million influenza vaccine doses^30^ were distributed by the NIP; one-third of these were enhanced doses for adults 65 years or older. AusVaxSafety 2018 surveillance captured approximately 1.8% of the total population (3.9 million people^31^) 65 years or older.

The NIP provides free influenza vaccines for all eligible Australian residents, based on age and risk factors for severe influenza.^32^ In 2018, influenza vaccines were funded for individuals 65 years or older, most Indigenous people, pregnant women, and individuals with chronic underlying medical conditions. Other licensed vaccines, which not supplied under the NIP, are also included in AusVaxSafety postmarketing surveillance, although they are not generally administered to large numbers of people.

Individuals vaccinated at participating sites or their caregivers respond yes or no to an initial SMS, automatically sent 3 to 5 days after immunization by 1 of 2 surveillance tools, SmartVax^33^ or Vaxtracker,^34^ asking whether any adverse events occurred after vaccination. Although 2 surveillance tools contribute to AusVaxSafety data, SmartVax is the predominant source, currently providing 99% of AusVaxSafety data, including all data in this analysis. SmartVax^35^ is installed at participating immunization sites and automatically extracts deidentified data on vaccination encounters from routinely used clinic software systems.

Respondents who reply yes to the initial question receive a second SMS asking whether they sought medical attention for the adverse event as well as a link to a short online survey asking them to specify the adverse event. Solicited adverse events include reports of any event (yes or no) and medical attention (our proxy for SAE as defined by the Australian Therapeutic Goods Administration^36^) (yes or no). Solicited adverse events include fever, injection site pain, injection site swelling or redness, tiredness, headache, sleep pattern change, irritability, rash, vomiting, diarrhea, rigors, nonresponsiveness or loss of consciousness, and seizures. Unsolicited symptoms can be detailed in free text.

Our analysis included all adults 65 years or older at who received any seasonal influenza vaccine between April 1 to August 31, 2018, and replied within 7 days to the initial SMS sent via the SmartVax tool by their participating AusVaxSafety immunization site. Persons who received 2 vaccine doses during the study period (such as select immunocompromised individuals) may be represented by more than 1 record.^37^

### Outcomes

Descriptive variables for respondents were summarized, including sex, Aboriginal and/or Torres Strait Islander (hereafter, *Indigenous*) status, age, timing or season of vaccination, vaccine brand and type, and concomitant vaccine administration (defined as any additional vaccine received at the same visit as the influenza vaccine). Indigenous status, when recorded, was provided by the individual at the practice level. AusVaxSafety assesses adverse event outcomes according to Indigenous status because Indigenous populations sometimes have different vaccination schedules and health outcomes.

### Statistical Analysis

Rates of adverse events were summarized by brand and type for HD-IIV3 (Fluzone High-Dose [Sanofi-Aventis]), aIIV3 (FLUAD [Seqirus]), and all standard nonadjuvanted QIIV brands in use (ie, Fluarix Tetra [GlaxoSmithKline], FluQuadri [Sanofi-Aventis], Afluria Quad [Seqirus], and Influvac Tetra [Mylan Health]) (Table 1), although these were not preferentially recommended in the study age group. Rates were compared using Pearson χ^2^ test. Characteristics of respondents who received HD-IIV3 and aIIV3 alone or with a concomitant vaccine were compared using Pearson χ^2^ test. *P* values were 2-sided, and statistical significance was set at *P* < .05.

**Table 1.** Seasonal Influenza Vaccines Available in Australia in 2018 by Brand and Recommended Age

Type	Brand	Name	Recommended age, y
Quadrivalent inactivated influenza vaccine	GlaxoSmithKline	Fluarix Tetra	≥3
	Sanofi-Aventis	FluQuadri Junior	<3 (6-35 mo )
		FluQuadri	≥3
	Seqirus	Afluria Quad	≥18
	Mylan Health	Influvac Tetra	≥18
High-dose trivalent inactivated influenza vaccine	Sanofi-Aventis	Fluzone High-Dose	≥65
Adjuvanted trivalent inactivated influenza vaccine	Seqirus	Fluad	≥65

Univariate analysis identified variables (chosen a priori) associated with adverse events and medical attention. Variables were included in a multivariable general linear model with Poisson distribution if they had a *P* < .25. Variables were retained in the multivariable model if they had a *P* < .05. All analysis was conducted in Stata statistical software version 14.2 (StataCorp). Analyses were conducted from September 1, 2018, to June 30, 2019.

## Results

### Study Cohort

From April 1 to August 31, 2018, 72 013 influenza vaccinations were administered to people 65 years or older at AusVaxSafety sentinel sites, and 50 134 individuals (69.6%; median [interquartile range] age, 71 [68-76] years; 27 056 [54.0%] women) responded to the initial SMS within 7 days of receiving it (Table 2).

**Table 2.** Overview of Participants 65 Years or Older in AusVaxSafety’s 2018 Influenza Vaccine Safety Surveillance

Variable	No. (%) (N = 50 134)
Women^a^	27 056 (54.0)
Aboriginal or Torres Strait Islander^b^	356 (0.8)
Age, median (IQR) [range], y	71 (68-76) [65-104]
Age group, y	
65-69	18 930 (37.8)
70-74	16 081 (32.1)
75-79	8756 (17.5)
80-84	4100 (8.2)
85-89	1568 (3.1)
90-94	572 (1.1)
95-99	116 (0.2)
100-104	11 (<0.1)
Vaccination period^c^	
Early vaccination season	35 211 (70.2)
Middle vaccination season	13 225 (26.9)
Late vaccination season	1698 (3.4)
Vaccine type (brand)	
aIIV3 (Fluad)	28 003 (55.9)
HD-IIV3 (Fluzone High-Dose)	19 306 (38.5)
QIIV	2208 (4.4)
FluQuadri	1223 (2.4)
Fluarix Tetra	534 (1.1)
Afluria Quad	425 (0.9)
Influvac Tetra	26 (0.1)
Other or unspecified	617 (1.2)
Receipt of ≥ 1 concomitant vaccine, No./Total No. (%)^d^	
Any	6176/50 134 (12.3)
PPSV23	4229/6176 (68.5)
Zoster	1370/6176 (22.2)
Tdap	361/6176 (5.9)

Abbreviations: aIIV3, adjuvanted trivalent inactivated influenza vaccine; HD-IIV3, high-dose trivalent inactivated influenza vaccine; IQR, interquartile range; PPSV23, 23-valent pneumococcal polysaccharide vaccine; QIIV, quadrivalent inactivated influenza vaccine; Tdap, tetanus diphtheria acellular pertussis.

^a^ Sex data were available for 50 108 participants.

^b^ Indigenous status data were available for 42 168 participants.

^c^ Early season was defined as April 2 through May 20, 2018; middle season, May 21 though July 8, 2018; late season, July 9 through September 2, 2018.

^d^ Concomitant vaccines may have been given alone with an influenza vaccine or in combination with each other and an influenza vaccine.

Most individuals received aIIV3 (28 003 individuals [55.9%]) or HD-IIV3 (19 306 individuals [38.5%]). A total of 2208 individuals (4.4%) received QIIVs. A total of 6176 encounters (12.3%) included at least 1 concomitant vaccine.

Recipients of aIIV3 and HD-IIV3 were similar in terms of sex and Indigenous status. Median (interquartile range) age was the same for both novel vaccines (aIIV3: 71 [68-76] years; HD-IIV3: 71 [68-75] years). More individuals received aIIV3 compared with HD-IIV3 early in the influenza vaccination season (22 336 individuals [67.0%] vs 10 998 individuals [33.0%]; *P* < .001). From middle to late vaccination season, HD-IIV3 was administered more frequently than aIIV3 (middle: 7305 individuals [58.5%] vs 5187 individuals [41.5%]; *P* < .001; late: 1003 individuals [67.6%] vs 480 individuals [32.4%]; *P* < .001). A greater proportion of individuals who received HD-IIV3 compared with individuals who received aIIV3 received at least 1 concomitant vaccine (2552 individuals [13.2%] vs 3241 individuals [11.6%]; *P* < .001). The most commonly received concomitant vaccine was 23-valent pneumococcal polysaccharide (PPSV23) (recommended at age ≥65 years and at least once 5 years later^37^), which was received by 1799 individuals who received HD-IIV3 (70.5%) and 2178 individuals who received aIIV3 (67.2%) (*P* = .007). Zoster vaccine (recommended at age 70-79 years) was the second most commonly received concomitant vaccine, received by 565 individuals who received HD-IIV3 (22.1%) and 742 individuals who received aIIV3 (22.9%) (*P* = .50).

### Outcomes

#### Rates of Any Adverse Event and Medical Attention

A total of 3684 individuals (7.4%) reported an adverse event, while 141 individuals (0.3%) reported seeking medical attention. More adverse events were reported by women than men (2357 individuals [8.7%] vs 1325 individuals [5.8%]; *P* < .001). A greater proportion of individuals who received HD-IIV3 reported any adverse events compared with individuals who received aIIV3 or any QIIV (1716 individuals [8.9%] vs 1796 individuals [6.4%] vs 140 individuals [6.3%]; *P* < .001). Adverse events were more commonly reported by the youngest individuals (1630 of 18 930 individuals aged 65-69 years [8.6%]; 1149 of 16 081 individuals aged 70-74 years [7.2%] years) and eldest individuals (1 of 11 individuals aged 100-104 years [9.1%]) (*P* < .001). Additionally, adverse events were more commonly reported by individuals who received vaccinations in the middle of vaccination season compared with those who received vaccinations early or late in the season (middle: 1071 of 13 225 individuals [8.1%]; early: 2484 of 35 211 individuals [7.1%]; late 129 of 1698 [7.6%]; *P* < .001). Despite these differences in reports of any adverse events, medical attention rates were similar by sex, brand or type, age group, and season.

#### Rates of Specific Adverse Events and Medical Attention by Vaccine Type

The most commonly reported solicited adverse event according to vaccine type were injection site pain (aIIV3: 350 of 26 880 individuals [1.3%]; HD-IIV3: 383 of 18 321 individuals [2.1%]; any QIIV: 24 of 2122 individuals [1.1%]; *P* < .001), injection site swelling or redness (aIIV3: 248 of 26 880 individuals [0.9%]; HD-IIV3: 256 of 18 321 individuals [1.4%]; any QIIV: 15 of 2122 individuals [0.7%]; *P* < .001), tiredness (aIIV3: 314 of 26 880 individuals [1.2%]; HD-IIV3: 347 of 18 321 individuals [1.9%]; any QIIV: 21 of 2122 individuals [1.0%]; *P* < .001), and headache (aIIV3: 242 of 26 880 individuals [0.9%]; HD-IIV3: 252 of 18 321 individuals [1.4%]; any QIIV: 23 of 2122 individuals [1.1%]; *P* < .001) (Table 3). For most solicited adverse events, rates associated with receipt of HD-IIV3 were higher than both those associated with aIIV3 or any QIIV receipt (Table 3). Similar rates for all 3 vaccine type categories were observed for reports of vomiting and diarrhea, as well as seizure and altered level of consciousness. Rates for seeking medical attention were similar among all 3 vaccine types (aIIV3: 80 of 27 665 individuals [0.3%]; HD-IIV3: 56 of 19 030 individuals [0.3%]; any QIIV: 5 of 2187 individuals [0.2%]; *P* = .86) (Table 3).

**Table 3.** Comparison of Crude Rates of Solicited Adverse Events for Individuals Who Received Influenza Vaccines

Type of adverse event	Respondents, No./Total (%)			*P* value
	aIIV3	HD-IIV3	Any QIIV	
Any	1796/28 003 (6.4)	1716/19 306 (8.9)	140/2211 (6.3)	<.001
Fever	164/26 880 (0.6)	195/18 321 (1.1)	14/2122 (0.7)	<.001
Injection site pain	350/26 880 (1.3)	383/18 321 (2.1)	24/2122 (1.1)	<.001
Injection site swelling or redness	248/26 880 (0.9)	256/18 321 (1.4)	15/2122 (0.7)	<.001
Rash	34/26 880 (0.1)	46/18 321 (0.3)	3/2122 (0.1)	.007
Tiredness	314/26 880 (1.2)	347/18 321 (1.9)	21/2122 (1.0)	<.001
Sleep pattern change	123/26 880 (0.5)	117/18 321 (0.6)	6/2122 (0.3)	.009
Headache	242/26 880 (0.9)	252/18 321 (1.4)	23/2122 (1.1)	<.001
Vomiting	21/26 880 (0.1)	24/18 321 (0.1)	3/2122 (0.1)	.19
Diarrhea	42/26 880 (0.2)	38/18 321 (0.2)	6/2122 (0.3)	.24
Rigors	26/26 880 (0.1)	50/18 321 (0.3)	2/2122 (0.1)	<.001
Irritability	60/26 880 (0.2)	50/18 321 (0.3)	0/2122	.04
Seizure	0/26 880	0/18 321	0/2122	NA
Altered level of consciousness	1/26 880 (<0.1)	0/18 321 (0)	0/2122	.68
Other^a^	197/26 880 (0.7)	221/18 321 (1.2)	15/2122 (0.7)	<.001
Medical advice	41/26 880 (0.2)	31/18 321 (0.2)	5/2122 (0.2)	.63
Medical attention	80/27 665 (0.3)	56/19 030 (0.3)	5/2187 (0.2)	.86

Abbreviations: aIIV3, adjuvanted trivalent inactivated influenza vaccine; HD-IIV3, high-dose trivalent inactivated influenza vaccine; NA, not applicable; QIIV, quadrivalent inactivated influenza vaccine.

^a^ Includes all unsolicited events. Participants could detail these in free text.

#### Rates of Any Adverse Event and Medical Attention by Concomitant Vaccination Status

Rates of adverse events were higher for individuals who received any concomitant vaccine compared with those who only received an influenza vaccine (906 of 6176 individuals [14.7%] vs 2778 of 43 958 individuals [6.3%]; *P* < .001) (Table 4). For individuals whose concomitant vaccine was PPSV23, rates were higher still (761 of 4229 individuals [18.0%]; *P* < .001) (Table 4). Rates of medical attention were higher for those receiving any concomitant vaccine compared with those who received only an influenza vaccine (46 of 6059 individuals [0.8%] vs 95 of 43 434 individuals [0.2%]; *P* < .001), and even higher for individuals whose concomitant vaccine was PPSV23 (40 of 4140 individuals [1.0%]; *P* < .001) (Table 4). Higher rates of any event and medical attention were reported for those receiving PPSV23 concomitantly with aIIV3 (any adverse event: 378 of 2178 individuals [17.4%]; *P* < .001; medical attention: 23 of 2133 individuals [1.1%]; *P* < .001) and HD-IIV3 (any adverse event: 341 of 1799 individuals [19.0%]; *P* < .001; medical attention: 14 of 1760 individuals [0.8%]; *P* < .001) (Table 4). Rates of adverse events and seeking medical attention were lower for individuals who received a zoster vaccine concomitantly (any adverse event: 112 of 1370 individuals [8.2%]; *P* = .23; medical attention: 6 of 1354 individuals [0.4%]; *P* = .27) than for those who received PPSV23 concomitantly (any adverse event: 761 of 4229 individuals [18.0%]; *P* < .001; medical attention: 40 of 4140 individuals [1.0%]; *P* < .001) (Table 4).

**Table 4.** Rates of Any Adverse Event and Medical Attention for Any Influenza Vaccine Stratified by Vaccine Type and According to Whether Influenza Vaccine Was Administered With a Concomitant Vaccine

Outcome	Respondents, No./Total (%)				
	Overall	Only influenza vaccine	Concomitant vaccine^a^		
			Any	PPSV23	Zoster
Any influenza vaccine					
Any adverse event	3684/50 134 (7.4)	2778/43 958 (6.3)	906/6176 (14.7)	761/4229 (18.0)	112/1370 (8.2)
Medical attention	141/49 493 (0.3)	95/43 434 (0.2)	46/6059 (0.8)	40/4140 (1.0)	6/1355 (0.5)
aIIV3					
Any adverse event	1796/28 003 (6.4)	1340/24 762 (5.4)	456/3241 (14.1)	378/2178 (17.4)	54/742 (7.3)
Medical attention	80/27 665 (0.3)	55/24 486 (0.2)	25/3179 (0.8)	23/2133 (1.1)	1/732 (0.1)
HD-IIV3					
Any adverse event	1716/19 306 (8.9)	1318/16 754 (7.9)	398/2552 (15.6)	341/1799 (19.0)	50/565 (8.9)
Medical attention	56/19 030 (0.3)	38/16 525 (0.2)	18/2505 (0.7)	14/1760 (0.8)	5/560 (0.9)

Abbreviations: aIIV3, adjuvanted trivalent inactivated influenza vaccine; HD-IIV3, high-dose trivalent inactivated influenza vaccine; PPSV23, 23-valent pneumococcal polysaccharide vaccine.

^a^ Participants may have received one or more concomitant vaccines. PPSV23 vaccine was the most commonly administered concomitant vaccine among those 65 years or older, administered to 68.5% of participants. Zoster vaccine was the second most commonly administered concomitant vaccine, administered to 22.2% of participants.

### Multivariable Analysis

Univariate analysis demonstrated that, compared with aIIV3, HD-IIV3 was associated with increased rates of any adverse event (risk ratio [RR], 1.1; 95% CI, 1.05-1.08; *P* < .001). This difference remained significant after adjusting for concomitant receipt of PPSV23, sex, and age group (adjusted RR, 1.1; 95% CI, 1.05-1.08; *P* < .001). Receipt of PPSV23 was also associated with increased reports of any adverse event (adjusted RR, 2.8; 95% CI, 2.58-3.00; *P* < .001). Univariate analysis demonstrated no significant association between brand and medical attention rates (RR, 1.0; 95% CI, 0.94-1.07; *P* = .92). However, receipt of PPSV23 in addition to HD-IIV3 was associated with an increased rate of medical attention (RR, 4.1; 95% CI, 2.82-6.00; *P* < .001).

## Discussion

This cohort study using active postmarketing surveillance directly compared the real-world safety of the enhanced vaccines HD-IIV3 and aIIV3 administered to Australians 65 years or older. Despite recent government and regulatory endorsement of the need for annual postmarketing influenza vaccine safety surveillance,^17,38^ systems are few and there remains a paucity of postmarketing safety data. The European Medicines Agency specifically states that active surveillance is the preferred method for quality postmarketing vaccine safety data.^17^ To our knowledge, our system is the largest global postmarketing active vaccine safety surveillance system in terms of participant numbers.

Our retrospective analysis included more than 50 000 individuals 65 years or older who received an influenza vaccine and responded to an SMS-based survey regarding adverse events experienced 3 to 5 days after vaccination. While near real-time safety monitoring in 2018 did not detect any safety signals, this end-of-season analysis found that individuals who received HD-IIV3 reported adverse events more commonly than those who received other influenza vaccines. However, this difference was not large, and absolute reported rates were not high. Reports consisted predominantly of nonserious events and did not vary greatly among brands. When adjusted for potential confounders, the increased risk of any adverse event associated with HD-IIV3 compared with aIIV3 was small. Our adjusted model demonstrated that younger age groups were significantly less likely than older age groups to report any adverse event, while women were more likely than men to report adverse events. These were likely associated with circumstantial or behavioral factors, such as the increased likelihood of women being attentive to health care concerns.^39^ Although individuals who received HD-IIV3 reported more injection site symptoms and fever, medical attention rates, our proxy for SAE, were low and similar for HD-IIV3 and aIIV3 at 0.3%. These empirical data demonstrated no unexpected burden on the health care system due to adverse events associated with influenza vaccines.

Randomized clinical trials of HD-IIV3 and aIIV3 have reported injection site pain in 36% of individuals who received HD-IIV3 (vs 24% of individuals who received standard TIIV) and 32% of individuals who received aIIV3 (vs 17% of individuals who received standard TIIV).^5,15,16^ In comparison, adverse event rates reported via our system were lower than these expected ranges. We believe the inherent differences in how adverse events were solicited and reported in these different contexts underpins this distinction. For example, in routine clinical practice, patients are advised on expected adverse events during the consent process and may not report trivial or expected adverse events; conversely, participants in randomized clinical trials are required to record any event in detail, sometimes with clinician oversight.

To our knowledge, no published randomized clinical trial has included direct comparison of these 2 enhanced influenza vaccines used in older adults, and only 1 other postmarketing comparison of HD-IIV3 and aIIV3 has been published. Using the US Vaccine Adverse Event Reporting System (VAERS), analysis of all spontaneously reported adverse events from July 2016 to June 2018 for aIIV3 were compared with HD-IIV3, as well as to standard TIIV and QIIV vaccines. Rates reported in VAERS were based on counts of specific adverse events reported out of the total number of events spontaneously reported for a vaccine type. Overall counts of the total of each vaccine type were small (aIIV3: 521 adverse events; HD-IIV3: 4383 adverse events; IIV3 and IIV4: 1095 adverse events),^40^ and interpretation of rates is constrained by traditional limitations of passive surveillance, including lack of denominator, variable reporting lags, and biases that may be introduced by underreporting, stimulated reporting, or incomplete reporting.^41,42^ However, in line with our results, VAERS demonstrated that injection site pain, swelling, and redness were more commonly reported by individuals who received HD-IIV3 compared with those who received aIIV3.^40^ In contrast to passive systems like VAERS, our participant-based monitoring allows for direct comparison of adverse events occurring within the first week of vaccination, underpinned by denominator data consisting of doses administered (recorded by brand in clinic software). Our large sentinel population enables detection of even minor differences in adverse event rates.

Our analysis, like previous data we have reported,^25,28^ demonstrated higher adverse event rates with receipt of a concomitant PPSV23. This outcome was observed for recipients of both enhanced vaccines. Increased local reactions associated with PPSV23, and in particular with revaccination, have been reported elsewhere.^43,44,45,46^ Receipt of PPSV23 concomitantly with influenza vaccine was also independently associated with increased medical attention rates compared with receipt of influenza vaccine alone.

### Limitations and Strengths

There are several limitations of our system and analysis. Although our system includes data specifying injection site arm, these data are currently not uniformly completed, and our ability to attribute a local reaction to a particular vaccine when more than 1 vaccine was received remains a limitation. Additionally, AusVaxSafety is designed to compile reports of adverse events that occur within days of vaccination and does not collect data on events occurring beyond this timeframe. The system in its current design does not detect very rare adverse events. In theory, given our large number of participants, we could identify very rare events for some vaccines or age groups. However, these unsolicited events would need to be detailed in free text by participants because they would not appear in our list of solicited events. Nevertheless, used in conjunction with Australia’s spontaneous adverse event reporting system, the Adverse Events Management System database administered by the Therapeutic Goods Administration, our active surveillance enhances Australia’s ability to potentially detect and investigate rare events.

Given the nature of active, participant-based surveillance, it is possible that some SAEs will go unreported if an individual is unable to report owing to the event or has died. Another expected limitation of adverse event surveillance is that not all events will be causally related to vaccination^47^; adverse event rates may be associated with illnesses with similar outcomes, such as fever from intercurrent viral illness, which cannot be differentiated from causally related events. Furthermore, our data rely on self- or caregiver-report of outcomes not clinically verified by a health professional and may be less objective for some adverse events compared with clinician-based reporting. While we have attempted to adjust for potential biases by reporting more objective outcomes, such as health care utilization and fever, this remains a limitation. Nevertheless, understanding individual perceptions of adverse events is valuable, and quantifying self-reported adverse events provides real-world safety data that are a window into individual perception of the safety of widely-administered vaccines.

Our study also has some strengths. Importantly, AusVaxSafety surveillance incorporates denominator data. Moreover, our system is timely and able to confirm the safety of each administered brand within weeks of the influenza vaccination program rollout. Since commencing influenza vaccine safety surveillance in 2014, our adverse event rates have, reassuringly, been stable and consistent across seasons.^25,26,27,28,48^ In addition to its timeliness, AusVaxSafety is able to be adapted quickly to conduct surveillance for new vaccines, and the system’s flexibility would facilitate its utility if a new pandemic vaccine were to be rolled out rapidly in the Australian population.

However, the essential strength of AusVaxSafety is its ability to enable clinicians to provide patients with a realistic portrait of adverse events that may occur after influenza vaccine receipt, based on adverse events reported by geographically and demographically similar people in near real time. This transparency, reported anecdotally by immunization stakeholders, including health care practitioners providing vaccine, empowers clinicians. We are currently evaluating the system to provide an objective assessment of this and to assess the extent to which this may influence decision-making and potentially result in greater confidence in, or uptake of, influenza and other vaccines.

## Conclusions

The findings of this large-scale participant-based postmarketing cohort study of the safety of 2 new enhanced influenza vaccines used in individuals 65 years or older provide reassuring near-real-time and cumulative data to inform and support confidence in ongoing vaccine use. As new influenza and other vaccines become available for use across the life course, new technologies and systematic surveillance methods, such as those used by AusVaxSafety and other active and enhanced passive surveillance initiatives in Canada, Italy, and elsewhere,^49,50,51^ should be adopted, where possible, to complement traditional passive surveillance for improved postmarket safety monitoring of all vaccines.
